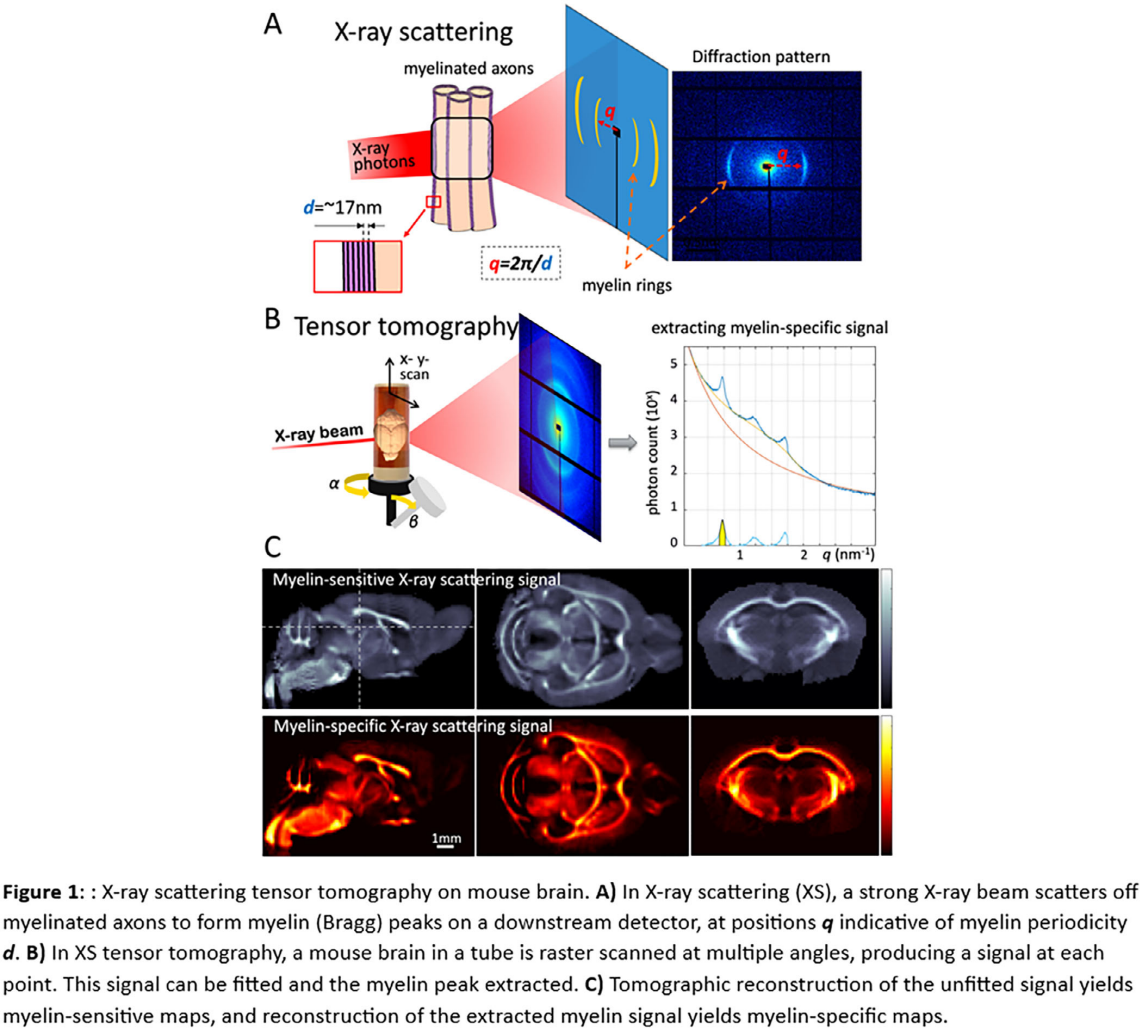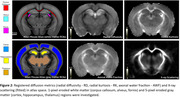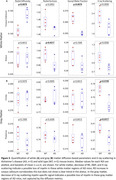# Myelin Changes in APPSweLon Mouse Model Detected by Diffusion MRI and X‐ray Scattering Tomography

**DOI:** 10.1002/alz70856_101618

**Published:** 2025-12-24

**Authors:** Andy Liu, Danielle Simmons, Frank Longo, Michael Zeineh, Marios Georgiadis

**Affiliations:** ^1^ Stanford University, Stanford, CA, USA

## Abstract

**Background:**

Myelin is involved in Alzheimer's Disease (AD), but its degeneration is difficult to quantify. Diffusion‐based MRI is sensitive to myelin changes, while X‐ray scattering can specifically image myelin. We use advanced diffusion metrics and X‐ray scattering to detect degenerative myelin changes in an *ex vivo* AD mouse model.

**Method:**

8‐month‐old male hAPP (APPSweLon mutation, *n* = 5) and wild‐type (WT, *n* = 5) mouse brains were perfusion‐fixed, and extracted. MRIs were acquired on a Bruker 7T (diffusion MRI with b=1,2,5,10ms/μm^2^, 100 directions, 200μm resolution). X‐ray scattering was acquired at the bioSAXS beamline of Stanford Synchrotron Radiation Lightsource (17KeV beam energy, 100ms exposure, 180 projections, 200μm resolution), Figure 1.

DESIGNER provided diffusion myelin‐sensitive metrics (mean/radial/axial diffusivities ‐MD/RD/AD, kurtoses ‐MK/RK/AK, axonal water fraction ‐AWF) (Figure 2). X‐ray scattering tomographic reconstruction provided quantitative myelin‐specific maps, Figure 1B,C. All images were non‐linearly registered to the Allen Atlas. Median values were computed for white matter (corpus callosum, alveus, fornix) and gray matter (cortex, thalamus, hippocampus), compared between groups using the Wilcoxon ranksum test.

**Results:**

In white matter, Figure 3A, the AD mice showed signs of myelin degeneration in diffusion MRI with significantly increased RD and reduced AWF in the corpus callosum and reduced RK in the alveus compared to controls, paralleled in X‐ray scattering with similar trends, corroborating possible white matter myelin degeneration. Fornix did not show any changes with either methods.

In the gray matter, Figure 3B, X‐ray detected significantly lower myelin levels in the cortex and the thalamus of AD mice compared to controls. Diffusion metrics, however, did not show any clear trends. Hippocampus did not show any changes with either methods.

**Conclusion:**

Imaging changes suggesting compromised myelin levels were detected in AD mice white matter and gray matter compared to controls. White matter changes were better detected with diffusion MR imaging. Gray matter changes were detected only using X‐ray scattering, not MRI, presumably due to the complexity of gray matter microstructure with many features besides myelin influencing diffusion MRI signal. X‐ray scattering may thus detect subtle degenerative myelin changes in regions where MRI methods show lower sensitivity.